# Optimal FSH usage in revascularization of allotransplanted ovarian tissue in mice

**DOI:** 10.1186/s13048-016-0299-7

**Published:** 2017-01-17

**Authors:** Wen-Zhi Ma, Xiao-Min Zheng, Chang-Chun Hei, Cheng-Jun Zhao, Sha-Sha Xie, Qing Chang, Yu-Fang Cai, Hua Jia, Xiu-Ying Pei, Yan-Rong Wang

**Affiliations:** 1 Key Laboratory of Fertility Preservation and Maintenance of Ministry of Education, Key Laboratory of Reproduction and Genetic of Ningxia Hui Autonomous Region, and Department of Anatomy, Histology and Embryology, Ningxia Medical University, Shengli street No.1160, Yinchuan, 750004 China; 2 The No, 1 People’s Hospital of xingtai, Hongxing street No.16, No, Xingtai, 054000 China

**Keywords:** FSH, Ovarian tissue, Revascularization, VEGF, bFGF

## Abstract

**Backgroud:**

Ovarian transplantation is a useful method for preserving the fertility of young women with cancer who undergo radiotherapy and chemotherapy. Follicle-stimulating hormone (FSH) is use to protect transplanted ovarian tissues from ischemia injury through promoting revascularization after transplantation, but the side effect of high level FSH is ovarian overstimulation leading to substantial follicular loss. In this study, we investigated the optimal usage of FSH on revascularization in the in vitro cultured ovarian tissues before and after transplantation.

**Results:**

FSH mainly exhibited an additive response in the gene and protein expression of vascular endothelial growth factor (VEGF), basic fibroblast growth factor (bFGF) and follicle stimulating hormone receptor (FSHR) with its raised concentrations (0.15 IU/ml, 0.30 IU/ml and 0.60 IU/ml) and prolonged treatment (3 h, 6 h, 12 h, 24 h). The concentrations with 0.60 IU/ml FSH could obviously promoted the expression of VEGF, bFGF and FSHR, but under this concentration FSH could also overstimulated the ovarian tissue leading to follicular loss. With the increase of culture time, the gene and protein expression of VEGF and bFGF both were up-regulated in all of the FSH added groups, but FSHR expression decreased when culture time exceeded 12 h. So we chose 0.30 IU/ml FSH added concentration and 6 h culture time as the FSH usage condition in functional revascularization verification experiment, and found that under this condition FSH promoted 2.5 times increase of vascular density in treated group than in control group after ovarian tissues transplantation.

**Conclusion:**

Ovarian intervention with 0.30 IU/ml FSH for 6 h is an optimal FSH usage condition which could accelerate the revascularization in the allotransplanted ovarian tissue and can not produce ovarian overstimulation.

## Backgroud

Preserving fertility has been considered to be one component of treatment in children, adolescents and women suffering from malignant tumors and in premenopausal patients [1–5]. Cryopreservation and transplantation of ovarian tissue have been employed for the preservation and restoration of women’s reproductive functions. This approach has provided hope for those who will lose their reproductive capacity.

More than 30 live human births have resulted from the transplantation of cryopreserved ovarian tissue [6], although various problems exist, such as the shortened lifespans of the transplanted ovaries, poor response to gonadotrophin and empty follicles without ovum [7–9], and ischemia caused by slow post-transplantation graft revascularization leading to substantial follicular loss. It is very important to restore ovarian function after transplantation of cryopreserved ovarian tissue, but the approaches to improve fertility restoration after this procedure are very limited. Treatment of ovarian tissue with vitamin E or gonadotrophins before transplantation could reduce ischemia and improve follicular survival in mice [10, 11]. Our previous study has shown that in vitro intervention with human menopausal gonadotrophin (HMG) before transplantation improved the blood supply reconstruction and survival of the autotransplanted follicles, which may be associated with increased VEGF expression[12].

Although there are a large number of studies confirming that HMG, FSH and luteinizing hormone (LH) can promote the expression of VEGF and increase the angiogenesis and the vascular permeability [13, 14], the optimum processing time and the concentration of FSH are still unknown. It is therefore very important to investigate the optimum therapeutic regimen of FSH in ovarian transplantation.

The present study was designed to investigate the optimal intervention by detecting the angiogenesis factor expression and vascular generation of ovarian tissue treated with different FSH concentrations and different durations of in vitro cultivation. We aimed to elucidate the therapeutic effects of FSH on cultured ovarian in clinical application of ovarian tissue transplantation.

## Methods

### Animals

The experiment was approved by the Ningxia Medical University Committee on the Use and Care of Animals. A total of 96 females, 5-week-old, Institute of Cancer Research (ICR) strain mice were used for this study. They were caged in a controlled environment at 20 °C with 12-h light/dark cycles. Standard mouse feed and water were provided ad libitum. All mice were allowed to acclimate to this environment for 1 week before initiation of the experiment. The mice were anaesthetized with an intraperitoneal injection of 0.3% napental (0.1 ml/10 g bodyweight; Beshide, Wuhan, China). Before surgery, the dorsolateral skin was shaved and antisepsis was obtained with 10% povidone iodine solution. Next, the bilateral ovaries were removed through small dorsolateral skin incisions, placed in Dulbecco’s phosphate-buffered solution (DPBS) (Sijiqing, Hangzhou, China) and were cut into two halves. Recipient mice kidneys were exposed in turn via two lateral incisions in the dorsolateral skin. Two fresh or FSH treated hemi-ovaries were immediately inserted at opposite poles of the two renal capsules of each recipient by creating a subcapsular pocket between the renal capsule and the renal parenchyma. After closing the wounds, the animals were returned to the isolators where they all made a full recovery.

### Culture of ovarian tissue

The bilateral ovaries of the ICR mice in diestrus were removed through small dorsolateral skin incisions placed in DPBS and were cut into two halves (2 × 1.5 × 1 mm). The ovarian tissues of each mouse were randomly divided into five groups: control group (not cultured); FSH 0.00 culture group (FSH 0.00-CG, no FSH cultured control); FSH 0.15 culture group (FSH 0.15-CG, cultured with FSH 0.15 IU/ml); FSH 0.30 culture group (FSH 0.30-CG, cultured with FSH 0.30 IU/ml) and FSH 0.60 culture group (FSH 0.60-CG, cultured with FSH 0.60 IU/ml). Ovarian tissue samples treated with different doses of FSH were cultured under 20% O_2_ and 5% CO_2_ at 37 °C for each of the culture durations (0 h, 3 h, 6 h, 12 h and 24 h).

### Immunohistochemistry

Ninety-six hemi-ovaries (from 24 mice, four hemi-ovaries from each mouse were divided with one hemi-ovary in each FSH concentration group: FSH 0.00-CG, FSH 0.15-CG, FSH 0.30-CG and FSH 0.60-CG) were processed routinely in 4% paraformaldehyde for 6 h after 3 h, 6 h, 12 h and 24 h in vitro culture. In control group (not cultured), six hemi-ovaries were removed from 6 mice without prior culture. Six ovarian tissues were used in each experimental group for each endpoint. The hemi-ovaries were processed routinely in 4% paraformaldehyde for 6 h. Paraffin sections (6 μm) were stained with EnVision two-step immunohistochemistry (Antibody Diagnostica, USA) and anti-rat VEGF, bFGF and FSHR polyclonal antibodies (1:100 dilution; Beshide, USA). In each experimental group for each endpoint, we used 6 hemi-ovaries. Each hemi-ovary was used to get one section. The section we used contained the biggest area from the cross-section of hemi-ovary. We stained the section to quantify immunohistochemical results. Three different high-power fields were chosen randomly, and the VEGF, bFGF and FSHR densities were estimated with integrated optical density. Two researchers assessed the results of the IHC analysis and obtained the same results. Cells staining positive for each of the markers were detected under light microscopy.

### Real-time PCR

On the basis of the immunohistochemistry experiment, we selected FSH 0.30-CG as the best treatment dose for the follow-up experiments. Total RNA was extracted from 60 hemi-ovaries (from 15 mice, four hemi-ovary from each mouse was placed in each FSH concentration group: FSH 0.00-CG and FSH 0.30-CG) using an AxyPrep kit (Chaoyan Biotech Co., Ltd, Shanghai, China) according to the manufacturer’s instructions after 0 h, 3 h, 6 h, 12 h and 24 h in culture. Six ovarian tissues were used in each experimental group for each endpoint. The real time quantitative polymerase chain reaction (RT-qPCR) procedure was carried out using ABI 7500fast and ABI 7500fast Optical System Software. RT-qPCR products were detected with SYBR Premix Ex Taq II. The primer sequences used are as follows: β-actin: 5′-TGGTTTTCTTGTTGCTCCCATA-3′, Rev, 5′-GGGTGCGGAGAAGGTTCAA-3′; VEGF: Fwd, 5′-CATCTTCAAGCCDTCCT GTGT-3′, Rev, 5′-CTCCAGGGCTTC ATCGTTACA-3′; bFGF: Fwd, 5′-CCCACCAGGCCACTTCAA-3′, Rev, 5′-GATGGAT GCGCAGGAAGAA-3′; FSHR: Fwd, 5′-TGTGCCAATCCTTTCCTCTATGC-3′, Rev, 5′-TTGTAAATCTGGGCTT GCACCTC-3′.

### Western blot

Immunoblotting analysis was performed as previously described[15]. Sixty hemi-ovaries (from 15 mice, four hemi-ovaries from each same mouse were included in the two different FSH concentration groups: FSH 0.00-CG, and FSH 0.30-CG) were lysed in radioimmunoprecipitation assay (RIPA) buffer containing a mixture of protease inhibitors on ice for 30 min after 0 h, 3 h, 6 h, 12 h and 24 h in culture. Six ovarian tissues were used in each experimental group for each endpoint. The samples were then separated on 12% sodium dodecyl sulfate (SDS) polyacrylamide gel with a 5% stacking gel under reducing conditions and transferred to the polyvinylidene difluoride (PVDF) membrane. The membranes were blocked with 5% non-fat dry milk for 1 h and probed with the first antibody at a concentration of 1 μg/ml for 1 h and then with a streptavidin-horseradish peroxidase-conjugated anti-rabbit antibody (ZhongBin GOLDENBRIDGE, China) at a dilution of 1:7000. The resulting signal was visualized using an ECL Detection kit (Thermo, USA) according to the manufacturer’s instructions. β-Actin was used as the reference. The results were analyzed using Image J Software.

### 2MD-FITC-Dextran perfusion of ovaries to assess revascularization

A total of 24 hemi-ovaries (from 12 donor mice, two hemi-ovaries from each mouse were placed in each of the two different FSH concentration groups: FSH 0.00-CG and FSH 0.30-CG) (six mice for each condition) were transplanted back to 12 recipient mice under the renal capsule after 3 h culture. For the control group, a total of 12 hemi-ovaries (from 6 donor mice) were transplanted to 6 recipient mice without prior culture. At 36 h after transplantation, the mice were given 10 mg/ml FITC conjugated Dextran with molecular weights of 2000000 daltons (2MD-FITC-Dextran) solution 0.1 ml by intravenous injection, with the exception of 6 mice in the control group. One hour later, the kidney with the implant was removed, fixed in 4% paraformaldehyde, and cut on a cryotome. In each experimental group for each endpoint we selected 3 sections from one hemi-ovaries. All frozen sections(40 μm) were mounted in 50% glycerin in water and viewed under a laser scanning confocal microscope. The green fluorescent of FITC conjugated dextran particles can be detected in renal capillaries and microvessels of the grafts after dextran perfusion. The area of the green fluorescent in microvessels of the ovarian grafts and the total area of grafts were counted by Image-Pro® Plus 6.0 (IPP 6.0) software. The vascular density was shown as the percentage of the ovarian microvessels area in the total area of ovarian grafts.

### Statistical analyses

All data were expressed as the mean ± SD. VEGF, bFGF, and the gene and protein expression of FSHR between groups (FSH 0.00-CG, FSH 0.15-CG, FSH 0.30-CG, and FSH 0.60-CG,) were analyzed with univariate analysis of variance. Significance was set at *P* < 0.05. Multiple comparisons of the data were performed using the Student-Newman-Keuls method.

## Results

### Expression and localization of VEGF, bFGF and FSHR in untreated and FSH-treated ovarian tissues detected by immunohistochemistry

**Fig. 1:**
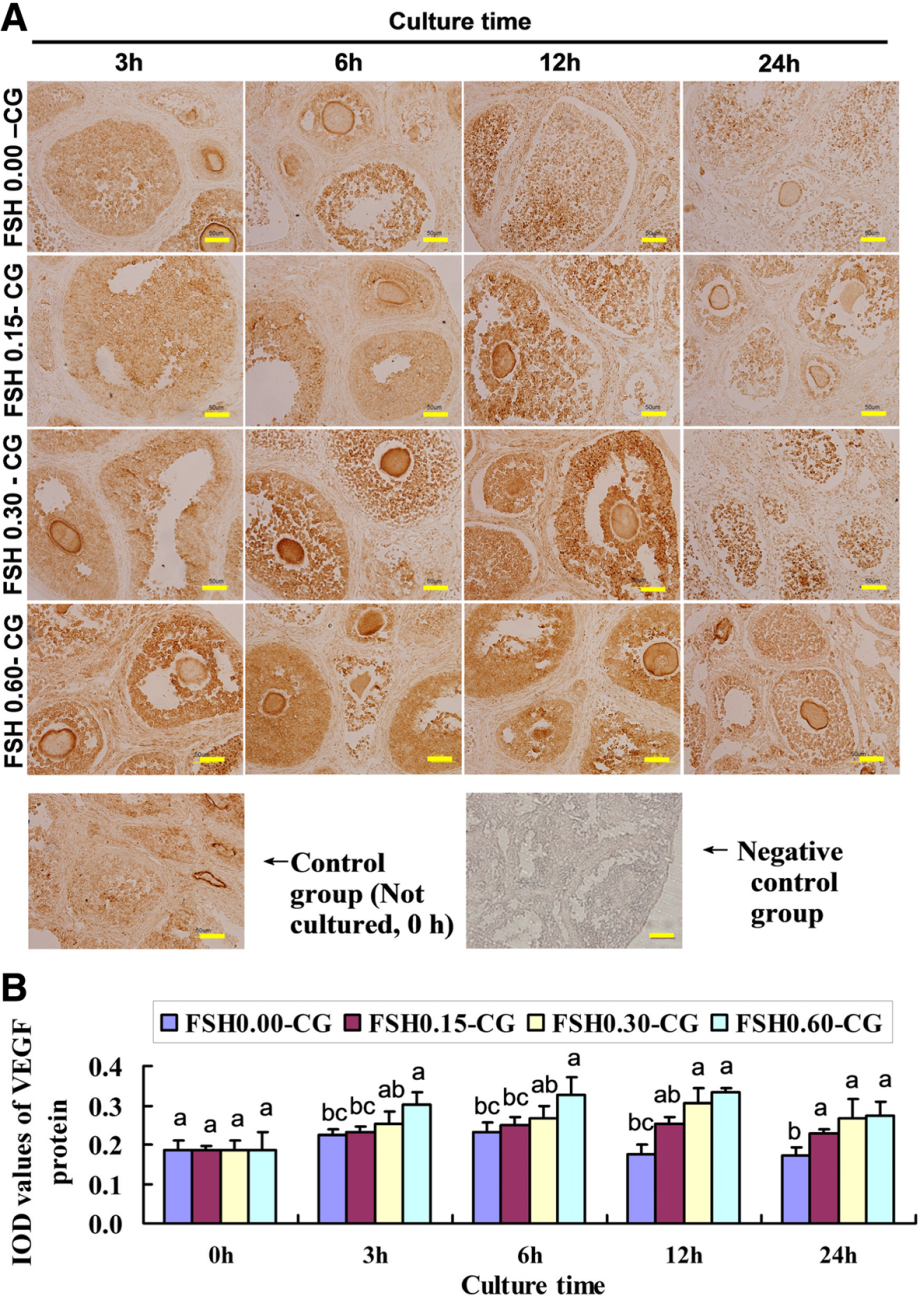
FSH up-regulated the protein expression of VEGF. **a** The immunohistochemical stainin of VEGF in four groups (FSH 0.00-CG, FSH 0.05-CG, FSH 0.30-CG, FSH 0.60-CG) with different FSH concentration at four time points(0 h,3 h, 6 h, 12 h, 24 h) and negative control staining for 24 h immunohistochemical endpoints. **b** Integrated optical density (IOD) of VEGF protein immunohistochemical stainin. Values with different superscripts in the same time point differ significantly (*P* < 0.05). Bar = 50 μm

**Fig. 2:**
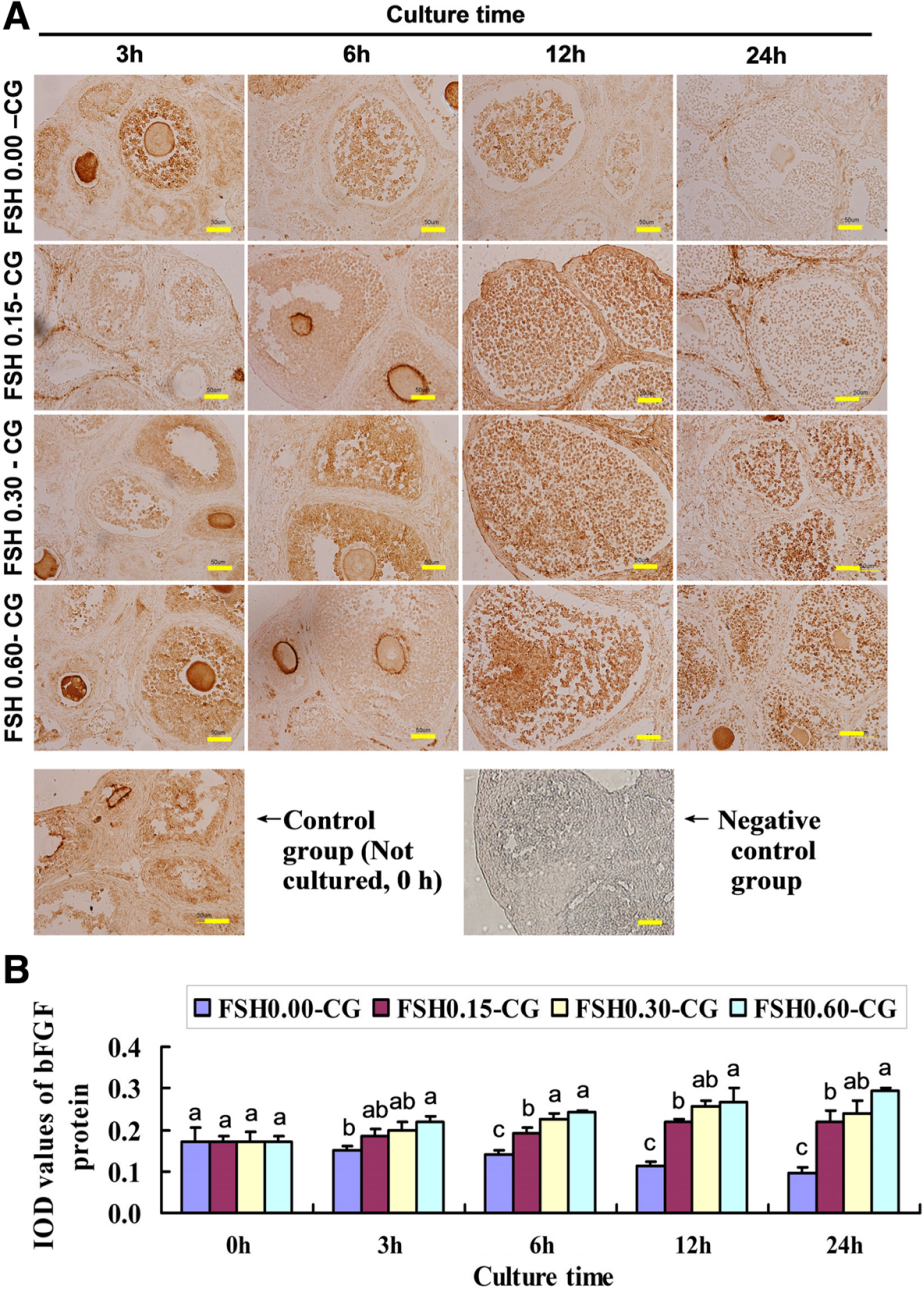
FSH up-regulated the protein expression of bFGF. **a** The immunohistochemical stainin of bFGF in four groups (FSH 0.00-CG, FSH 0.05-CG, FSH 0.30-CG, FSH 0.60-CG) with different FSH concentration at four time points(0 h,3 h, 6 h, 12 h, 24 h) and negative control staining for 24 h immunohistochemical endpoints. **b** Integrated optical density (IOD) of bFGF protein protein immunohistochemical stainin. Values with different superscripts in the same time point differ significantly (*P* < 0.05). Bar = 50 μm

**Fig. 3:**
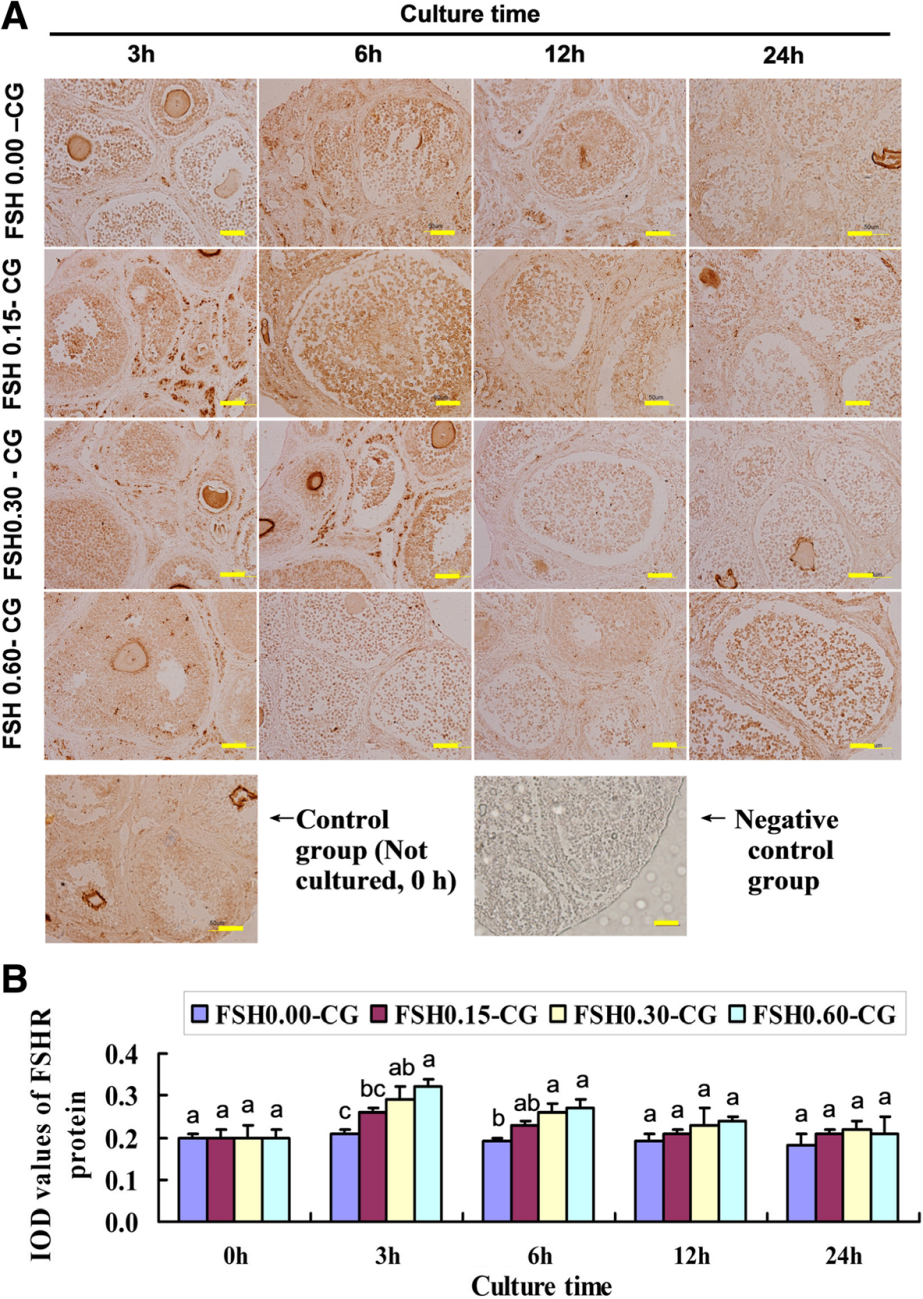
FSH up-regulated the protein expression of FSHR. **a** The immunohistochemical stainin of FSHR in four groups (FSH 0.00-CG, FSH 0.05-CG, FSH 0.30-CG, FSH 0.60-CG) with different FSH concentration at four time points(0 h,3 h, 6 h, 12 h, 24 h) and negative control staining for 24 h immunohistochemical endpoints. **b** Integrated optical density (IOD) of FSH protein immunohistochemical stainin. Values with different superscripts in the same time point differ significantly (*P* < 0.05). Bar = 50 μm

The proteins of VEGF (Fig. 1), bFGF (Fig. 2) and FSHR (Fig. 3) were mainly expressed in the cytoplasm of the granulosa cells of the large follicle. VEGF and bFGF were also expressed in theca cells. Interestingly, the expression levels of VEGF, bFGF and FSHR increased after FSH treatment, and the expression of these proteins is FSH dose dependent. The expressions of VEGF and bFGF in the FSH 0.60-CG were significantly higher (*P* < 0.05) than those in FSH 0.00-CG at all of the culture time points. The expression level of FSHR protein in FSH 0.60-CG and 0.30-CG significantly higher (*P* < 0.05) than those in FSH 0.00-CG at 3 h and 6 h, and the expression in the FSH-added group(FSH 0.60-CG and 0.30-CG) was not statistically different from FSH 0.00-CG at 12 h and 24 h after FSH treatment. While the expressions of VEGF, bFGF and FSHR in the FSH 0.60-CG group were no significantly different (*P* < 0.05) from FSH 0.30-CG at all of the culture time points. Our previous study have shown that FSH treatment with high concentration of 0.60 IU/mL could induced ovarian overstimulation. Based on these experimental results and our previous study, we selected 0.30 IU/mL FSH as the treatment concentration used in follow-up experiments to quantitatively detect VEGF, bFGF and FSHR gene and protein temporal expression.

### Gene expression of VEGF, bFGF and FSHR in FSH-treated ovaries

**Fig. 4:**
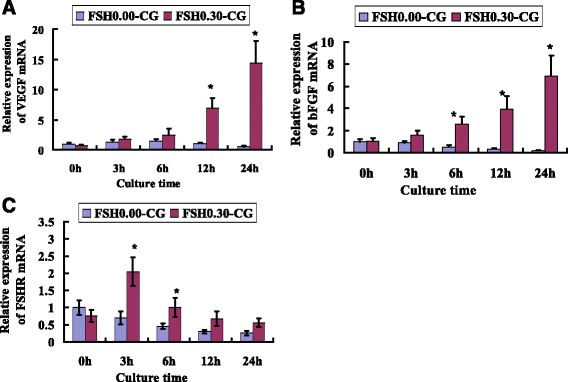
FSH up-regulated the mRNA expression of VEGF, bFGF and FSHR. Real-time PCR analysis of VEGF (**a**), bFGF (**b**) and FSHR (**c**) in FSH 0.00-CG and FSH 0.30-CG. The data were presented as fold changes relative to FSH 0.00-CG at 0 h. Asterisk (*) indicates a significant increase between FSH 0.00-CG and FSH 0.30-CG at the same time point (*P* < 0.05)

Real-time quantitative PCR was carried out to further confirm the increased expression of VEGF, bFGF and FSHR in the FSH intervention group at different culture times. The results of qPCR showed that the expression level of these mRNA is time dependent. VEGF mRNA was significantly higher (*P* < 0.05) at 12 h and 24 h compared to the expression in FSH 0.00-CG at the same time points (Fig. 4). The expression of VEGF mRNA was increased 26.27 times in the FSH 0.30-CG group compared with that in FSH 0.00-CG after 24 h of culture. The expression of bFGF mRNA was significantly higher (*P* < 0.05) in the FSH 0.30-CG at 6 h, 12 h and 24 h compared to the expression in FSH 0.00-CG at the same time points (Fig. 4). The expression of bFGF mRNA was increased 33.2 times in the FSH 0.30-CG group than that in FSH 0.00-CG after 24 h of culture. The expression of FSHR mRNA was significantly higher (*P* < 0.05) at 3 h and 6 h than that in the FSH 0.00-CG group at the same time points. The expression of FSHR mRNA was increased 2.94 times in the FSH 0.30-CG group than that in FSH 0.00-CG after 3 h of culture and gradually diminished with culture time, while the expressions of VEGF, bFGF and FSHR mRNA gradually diminished with culture time in FSH 0.00-CG.

### Protein expression of VEGF, bFGF and FSHR in FSH-treated ovaries

**Fig. 5:**
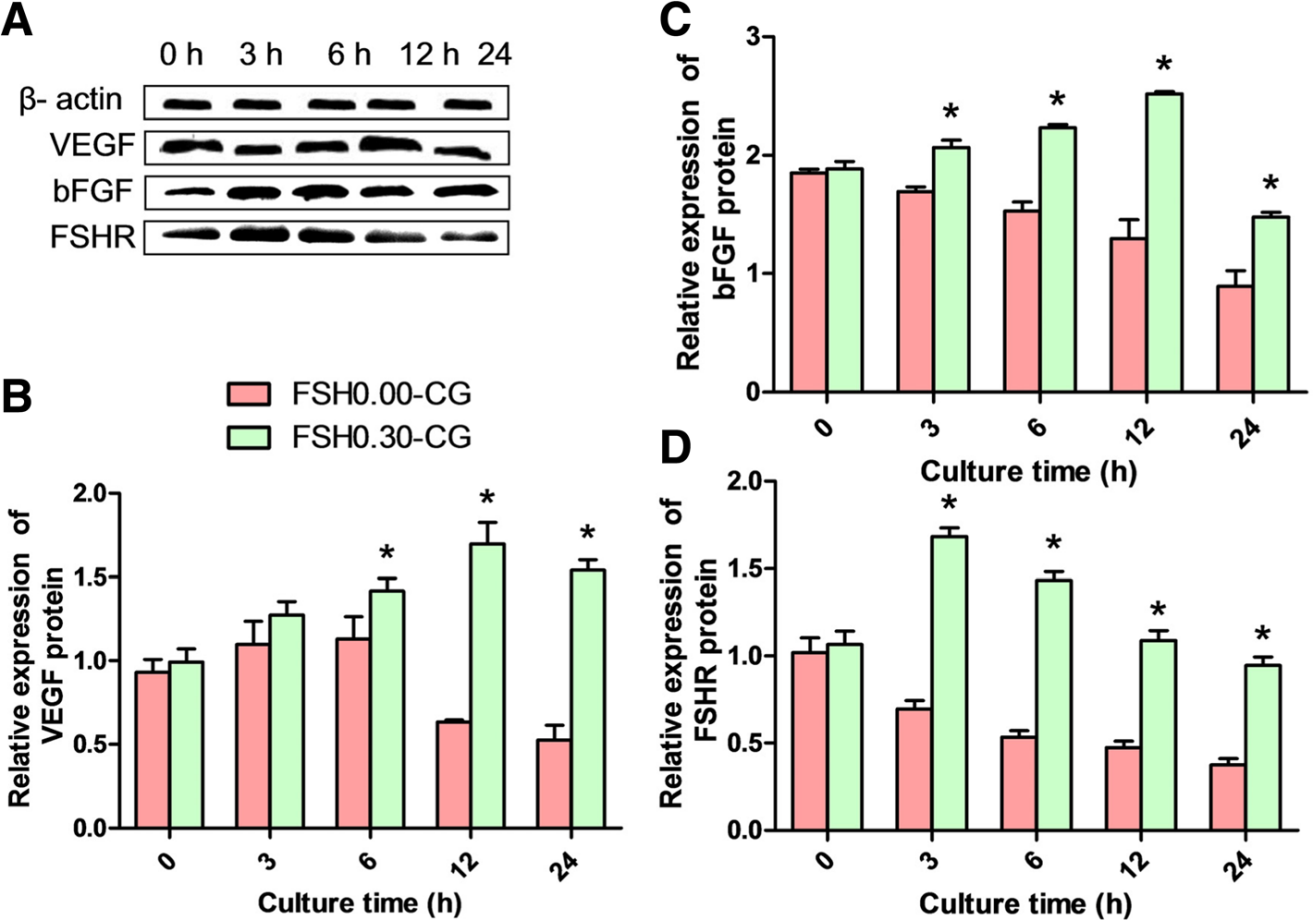
FSH up-regulated the relative protein levels of VEGF, bFGF and FSHR. The relative protein expression of VEGF (**a**, **b**), bFGF (**a**, **c**) and FSHR (**a**, **d**) in FSH 0.00-CG and FSH 0.30-CG detected by western blot. The protein levels of VEGF and bFGF based on densitometry units showed gradual increase from 0 h to 12 h in culture in FSH 0.30-CG. The protein level of FSHR based on densitometry units showed a significant increase from 3 h to 6 h in FSH 0.30-CG. The relative protein levels of VEGF, bFGF and FSHR decreased gradually from 6 h to 24 h in culture in FSH 0.00-CG. Asterisk (*) indicates a significant increase between FSH 0.00-CG and FSH 0.30-CG at the same time point (*P* < 0.05)

The expression levels of VEGF, bFGF and FSHR were then submitted to western blot analysis to further evaluate their protein levels in the FSH intervention group and the control group. The results showed that the protein level of VEGF based on densitometry units significantly (*P* < 0.05) increased during 6 h to 24 h of culture in FSH 0.30-CG compared with FSH 0.00-CG (Fig. 5). The protein level of bFGF based on densitometry units was significantly higher (*P* < 0.05) at 3 h, 6 h, 12 h and 24 h of culture compared to the expression in the FSH 0.00-CG group. The protein level of FSHR based on densitometry units showed a significant increase (*P* < 0.05) in 3 h and 6 h of culture in FSH 0.30-CG compared to FSH 0.00-CG. Although VEGF temporarily increased at 3 h and 6 h of in vitro culture., the expression of VEGF, bFGF and FSHR protein gradually diminished with culture time in FSH 0.00-CG.

### Blood perfusion in transplanted grafts

**Fig. 6:**
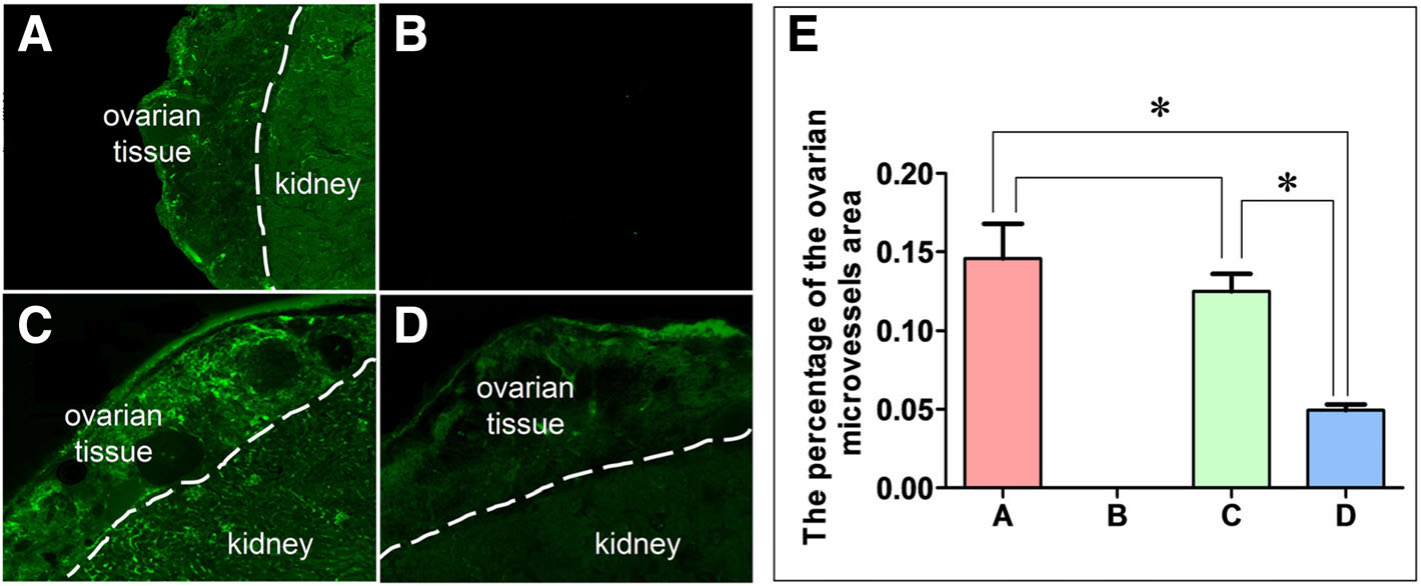
FSH accelerate bloodstream reperfusion in transplanted ovarian tissues. A control group (not cultured): (**a**) number of 2MD-FITC-Dextran granules in ovarian blood vessels 36 h after transplantation of fresh ovaries; (**b**) negative control (no 2MD-FITC-Dextran injected control): There were no 2MD-FITC -Dextran granules in the ovarian tissue at 36 h after transplantation of ovaries which treated with 0.30 IU/ml FSH for 6 h; (**c**) culture with 0.30 IU/ml FSH: Numerous 2MD-FITC-Dextran granules in ovarian blood vessels 36 h after transplantation of ovaries which treated with 0.30 IU/ml FSH for 6 h; (**d**) culture with 0.00 IU/ml FSH: Few 2MD-FITC-Dextran granules in the ovarian blood vessels 36 h after transplantation of ovaries which treated with 0.00 IU/ml FSH for 6 h; (**e**). Mean vascular density of transplanted ovarian tissues. Asterisk (*) indicates a significant increase between the two groups (*P* < 0.05) (×200)

To further investigate the effect of FSH on the neovascularization in the transplanted grafts, 2MD-FITC-Dextran perfusion was used. Because VEGF and bFGF protein expression were higher after 12 h FSH treatment, and FSHR protein expression was higher after 3 h FSH treatment. While after 12 h culture FSH lead to overstimulation of ovarian tissues, and the gene and protein expression of FSHR were also began to decrease after this time point. So we selected the ovarian tissue that were cultured for 6 h with FSH to transplant into the recipient. The green particles can be observed in renal capillaries and microvessels of the grafts after 2MD-FITC-Dextran perfusion. Thirty-six hours after transplantation, the blood vessels could be detected in all the groups, whereas no signal was detected in the no 2MD-FITC-Dextran injected negative control group. The vascular density in FSH 0.30-CG was significantly higher (*P* < 0.05) than that in FSH 0.00-CG, while there was no significant difference between the two groups. In addition, the green particle-filled blood vessels in FSH 0.30-CG were more centrally located within the ovarian tissue than those in FSH 0. 00-CG and (Fig. 6).

## Discussion

Although ovarian transplantation is a very useful method to preserve the fertility of young women who suffered from cancer and have been treated with radiotherapy and chemotherapy, hypoxic-ischemic damage is the major challenge in ovarian tissue transplantation [16, 17]. Vascular remodeling is very important for ovarian tissue survival and functional recovery after transplantation. It has been noted that gonadotrophins, such as HMG, LH and FSH, are survival factors for follicles [12, 16, 17]. It has also been reported that the administration of FSH after the transplantation of ovarian tissue is beneficial to the reconstruction of blood vessels in ovarian tissue; however, gonadotrophin stimulation with long time leads to loss of follicles [18–21]. The optimum concentration and duration of FSH intervention are extremely important to promote angiogenesis. Based on these findings, the transient intervention with the proper dose and duration of FSH during in vitro culture of ovarian tissue could promote the revascularization after ovarian heterotopic transplantation.

The immunohistochemical results showed that VEGF is mainly expressed in the cytoplasm of ovarian granulosa cell, theca cell and oocyte. Consisted with our results, previous studies revealed that VEGF is expressed in oocyte of ostrich [22], rat [23], ovine [24], porcine [25] and human [26]. In the intervention groups, with the increase in FSH concentrations, the expression of VEGF increased gradually. These results indicated that VEGF expression was dose dependent. VEGF has been regarded as a target gene of FSH [27], and the expression of VEGF is promoted by FSH [28, 29]. New evidence has shown that VEGF has protective effects on cell survival [30]. The expression of VEGF mRNA and protein in the higher FSH concentration group (cultured with FSH 0.30 IU/ml) was significantly higher than in the no FSH addition group. In addition, ovaries cultured with the highest concentration of FSH for 3 h yielded more exudates between the host renal capsule and donor 36 h after graft transplantation and had fewer follicles 1 month after transplantation. This finding may be the result of excessive activation of the ovaries by the higher concentrations of FSH, causing follicular consumption and leading to ovarian reserve loss. The longer duration of FSH stimulation can increase the gene and protein expression of angiogenesis factors, which is helpful for the establishment of the revascularization after transplantation. Previous studies have shown that transplanted ovaries with an extended duration of gonadotrophin intervention may lead to significant loss of primordial follicles [19]. Therefore, the in vitro ovarian culture time with FSH should be appropriate.

bFGF is important for the promotion of cell mitosis. In studies, we found bFGF promotes new blood vessel generation and accelerates the healing of damaged tissue. Ovaries of women of reproductive age with normal ovarian function have been shown to express bFGF in the corpus luteum, granulosa cells [31]. Ovaries of the ostrich (struthio camelus) have been shown to express bFGF in oocyte [22]. Sheep [32] and rat [33] oocytes also show positive staining for bFGF. However, with aging, ovarian function declines and bFGF expression gradually reduces. bFGF can stimulate the rapid formation of a capillary network around a preovulatory follicle and induce vascular change during the early corpus luteum formation. Our previous studies have confirmed that FSH up-regulates bFGF gene expression, although little data exists with regard to whether the amount of FSH influences the expression of bFGF. Our present results showed that FSH can up-regulate the expression of bFGF mRNA and protein and that the expressions are dose dependent with the increase in FSH concentration. This study further found that bFGF protein expression increased with prolonged FSH culture duration. Our studies provide a scientific basis for in vitro FSH intervention to promote vascular remodeling.

In this study, we found that VEGF and bFGF gene expressions were increased with the duration of FSH culture. The amount of mRNA of VEGF and bFGF reached their peak 24 h after ovarian culture with FSH, which was statistically significant compared to the no-FSH added group (*P* < 0.05). VEGF and bFGF protein expressions were also increased with the extension of the incubation time in the culture medium with FSH. The amount VEGF and bFGF proteins reached their peaks 12 h after ovarian culture with FSH, while the ovarian expression peak of FSHR mRNA and protein appeared at 3 h after culture with FSH. Previous studies showed that when endothelial cells are cultured in vitro using medium supplemented with growth factor bFGF, the expression of VEGF was increased. If the expression of VEGF was blocked, bFGF therefore cannot induce endothelial cells to form blood vessels. Thus, endothelial cell angiogenesis was promoted by VEGF, which was up-regulated by bFGF [34]. The bFGF was transported to the nucleus and enhanced VEGF transcription after binding with its receptors. Additionally, bFGF up-regulated the expression of VEGF, which sped up the process of wound healing [35]. Therefore, bFGF and VEGF also have synergy in the process of angiogenesis. Ovarian tissue samples treated with different doses of FSH were cultured under 20% O_2_ and 5% CO_2_, in which the ovarian tissue is under the normal condition and it is not in a hypoxia condition. Hypoxia can usually induce HIF1a expression, and then up-regulate VEGF expression which promote angiogenesis. Hence, the up-regulated expression of VEGF was induced by FSH. Our experimental results show that the expression of the FSHR gene and protein reached a peak 3 h after FSH treatment, which amplified the limited FSH effects.

In the current study, we transplanted half of the ovaries that had been cultured with the concentrations of 0.30 IU/ml FSH in vitro for 6 h back to the recipient mouse body and observed the vascularization process at 36 h after transplantation. The results demonstrated blood perfusion from host to implanted tissue appeared in the FSH 0.30-CG. Meanwhile, the expression of VEGF and bFGF in the FSH 0.30-CG was significantly higher than in the other groups. This study, in addition to previous studies, have suggested that the expression of VEGF and bFGF were regulated by FSH during the in vitro culture of the ovary[36–38]. The up-regulation of VEGF and bFGF is helpful for the formation of novel blood vessels during ovary transplantation. It also increases the rate of ovary transplantation. These results indicated that the blood perfusion between the graft and the host was related to the addition of FSH.

## Conclusion

These findings demonstrate that ovarian intervention with 0.30 IU/ml FSH for 6 h is an optimal FSH usage condition which could accelerate the revascularization in the allotransplanted ovarian tissue and can not produce ovarian overstimulation in mice.

## Abbreviations

2MD-FITC-Dextran: FITC conjugated dextran with molecular weights of 2000000 daltons
bFGF: Basic fibroblast growth factor
DPBS: Dulbecco’s phosphate-buffered solution
FSH 0.00-CG: FSH 0.00 culture group
FSH 0.15-CG: FSH 0.15 culture group
FSH 0.30-CG: FSH 0.30 culture group
FSH 0.60-CG: FSH 0.60 culture group
FSH: Follicle-stimulating hormone
FSHR: Follicle stimulating hormone receptor
HMG: Human menopausal gonadotrophin
ICR: Institute of Cancer Research
LH: Luteinizing hormone
PVDF: Polyvinylidene difluoride
RT-qPCR: The real time quantitative polymerase chain reaction
VEGF: Vascular endothelial growth factor
